# Business forecasting methods: Impressive advances, lagging implementation

**DOI:** 10.1371/journal.pone.0295693

**Published:** 2023-12-14

**Authors:** Paul Goodwin, Jim Hoover, Spyros Makridakis, Fotios Petropoulos, Len Tashman

**Affiliations:** 1 School of Management, University of Bath, Bath, United Kingdom; 2 Warrington College of Business, University of Florida, Gainesville, FL, United States of America; 3 Makridakis Open Forecasting Center, University of Nicosia, Nicosia, Cyprus; 4 International Institute of Forecasters, Medford, MA, United States of America; National University of Sciences and Technology, PAKISTAN

## Abstract

Reliable forecasts are key to decisions in areas ranging from supply chain management to capacity planning in service industries. It is encouraging then that recent decades have seen dramatic advances in forecasting methods which have the potential to significantly increase forecast accuracy and improve operational and financial performance. However, despite their benefits, we have evidence that many organizations have failed to take up systematic forecasting methods. In this paper, we provide an overview of recent advances in forecasting and then use a combination of survey data and in-depth semi-structured interviews with forecasters to investigate reasons for the low rate of adoption. Finally, we identify pathways that could lead to the greater and more widespread use of systematic forecasting methods.

## 1 Introduction

The effects of the COVID pandemic and the Ukraine war have further called into question the role of forecasters and the benefits of their prognostications. Even in kinder times their efforts have been subjected to skepticism and, frequently, outright hostility. “My year-end forecast: there is no future for prediction,” wrote Dominic Lawson in Britain’s *Sunday Times* in 2014, annoyed that forecasters had failed to anticipate a 40% drop in oil prices. But there are many conditions where forecasts have proven to be acceptably reliable. Five-day weather forecasts are now reasonably accurate. Tetlock and Gardner’s *Superforecasters* [1] has shown that even open-minded nonspecialists (“foxes”) working together systematically can provide superior forecasts of world events.

In addition, recent years have seen significant advances in forecasting techniques so that key decisions now have the potential to be based on more accurate assessments of future prospects. Some companies have experienced considerable cost savings by adopting these methods—reducing inventory levels and improving customer-service levels simultaneously. However, there is evidence that these companies are in the minority and most are not exploiting the benefits that reliable and systematic forecasts can bring. In this paper we use a combination of survey data and in-depth interviews with forecasters to establish reasons for this low rate of adoption. We then suggest future pathways that could lead to a more widespread take up of systematic methods.

## 2 The necessity of forecasts

For most organizations, avoidance of forecasting is not an option. Decisions about future production levels, staff recruitment, service capacity, or resource allocation between competing projects—these all require predictions of what tomorrow might bring.

Moreover, these predictions must be trusted and reliable. Their rationale and limitations need to be understood and accepted by decision makers. They need to be free of political bias and wishful thinking. And they need to make the best use of available information and expert knowledge subject to cost-effectiveness. To accomplish all this requires skilled forecasting personnel who can make effective use of forecasting software and have the communications skills to provide explanations tailored to the users and decisions they are intended to support.

## 3 Noteworthy methodological advances

Recent decades have witnessed dramatic enhancements in forecasting methods. Forecasting today is a respected academic field and a valued profession in organizations, with new ideas and methods constantly emerging.

The forecaster’s toolbox, with its earlier methods rooted in statistics, operations research, and econometrics, has been expanded to encompass an array of new techniques.2 These include:

- Models based on machine learning (ML), such as neural networks and decision trees;
- Hybrids of statistical and ML models, one of which was the top performer in the M5 Competition [2–4];
- Automatic method-selection protocols such as ETS, which are now embedded in commercial and open-source software;
- New techniques for modeling promotions, other discrete events, and rare but possibly recurring events;
- New hierarchical reconciliation procedures for ensuring coherence in product and temporal hierarchies;
- Enhanced forecast evaluation designs and metrics;
- Techniques for supporting and enhancing the role of judgment in forecasting.

Ample evidence has emerged that these methodological advances have improved forecast accuracy and the calibration of the uncertainty behind the forecasts. Today, an organization can utilize a broad spectrum of methods and systems enabling it to determine optimal models for its data.

## 4 Systematic forecasting

We define *systematic forecasting* as the application of appropriate statistical and algorithmic methods to available historical data, while allowing for justifiable judgmental interventions when information relating to novel or special circumstances must be considered. Such a process normally requires dedicated forecasting software, the collection and cleaning of relevant data, and continuous monitoring of forecast accuracy. Note that our definition does not rule out application of judgment per se. It can encompass an array of structured judgmental procedures such as Delphi processes and scenario development, especially in situations such as new-product forecasting where historical data is limited or unavailable.

Systematic forecasting is not crystal-ball gazing. The methods do not possess prophetic powers. Instead, their algorithms seek to identify patterns and relationships in relevant data, filtering out random perturbations and producing assessments of uncertainty. These patterns are then projected ahead on the assumption that they will continue. Where this assumption is unlikely to be valid, as during the COVID pandemic, experts’ judgments must adjust or replace these projections, though this should be carried out carefully given the possible contaminating effects of cognitive and motivational biases.

Extensive experience has shown that the use of systematic forecasting methods (SFMs) are less susceptible to bias, and are superior in accuracy [5, 6]. Given these significant benefits, we should ask: To what extent are organizations recognizing these benefits and harnessing the most appropriate technology? While supersized enterprises in retail (Walmart, Target), in cloud computing and search (AWS, Google), and in manufacturing and logistics (automotive, electronics) have successfully exploited SFMs, anecdotal evidence suggests that many organizations have refrained from adopting them. Survey evidence backs this up. Over 25% of company forecasts were based on judgment alone in surveys by Fildes and Goodwin [7] and Weller and Crone [8].

Our mission was to gather data about the diffusion of SFMs, and establish what is preventing nonadopting firms from exploiting their potential.

## 5 Why are SFMs missing in action?

Our findings are based on survey responses from 370 managers in organizations of different sizes, and 20 in-depth, semistructured interviews with practitioners (2/3) and consultants (1/3). This survey was conducted in two groups. The first survey was sent to over 5,000 small business owners and received 179 useable responses. The small business owner group was chosen because previous consulting experience had shown them much less likely to use systematic forecasting methods. Many of these firms were managers or owners who did not have forecasting staff. The second version of the survey was sent to primarily medium and large firms. Over 5,000 surveys were emailed to these firms and managers. Many of the 191 responses in the second survey came from the position of CEO/COO/Owner/Executive or those directly involved in the forecasting or demand planning processes. Together these two surveys received a combined total of 370 responses reporting firm size in the categories of large (over 500 employees: N = 99); medium (100–500 employees: N = 35); small (25–100 employees: N = 81); and micro (less than 25 employees: N = 155). The interviews supplemented the survey by exploring in detail how current forecasting practices depart from appropriate application of SFMs.

For the interview data, which was organised by the first and fourth authors of this paper, ethical approval was granted by the University of Bath (no S21–053). Consent was informed by written methods (signature). Data collection was performed from May 2021 until October 2021.

For the first survey, which was organised by the second author of this article, ethical approval was granted by the University of Florida, (no IRB202002439). Consent was informed by digital methods. This approval was granted with “Exempt” status meaning that it was categorized as very low risk (since it was a survey and followed the Opt Out directions of the University). Data collection was performed in October 2020.

The second survey was conducted by the publication Foresight of the International Institute of Forecasters. The survey contained the same questions as the first survey (with a few slight modifications). The survey was conducted commercially using multiple email listings including. Because this survey was conducted commercially by Foresight, it did not go through an additional ethical approval. Consent was informed by digital methods. Data collection was performed in June 2021.

Our survey began by asking: *We define systematic forecasting as the application of statistical or machine learning (ML) methods to your available historical data, while allowing for judgmental inputs and adjustments. To what extent does your firm make use of systematic forecasting methods to generate forecasts?*

**Fig 1:**
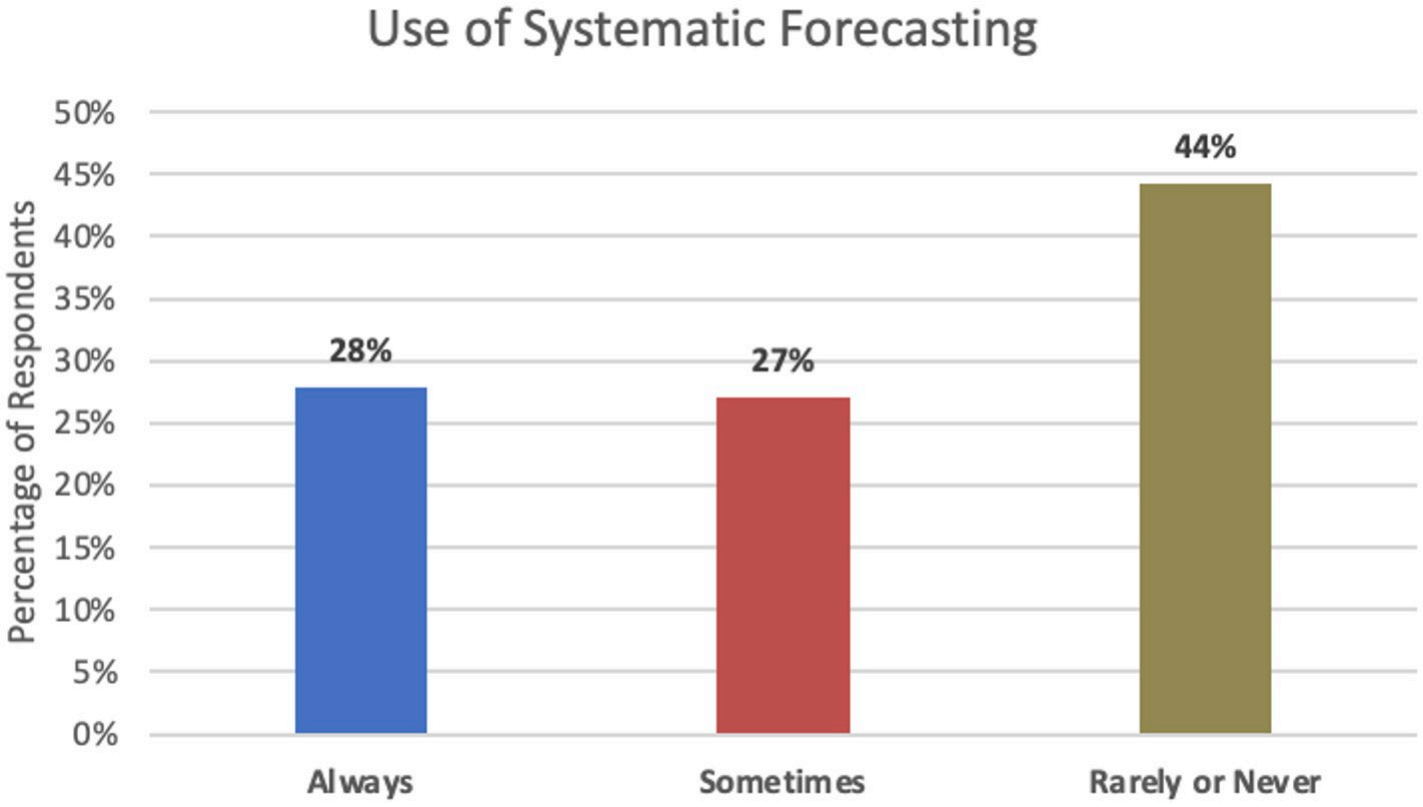
Frequency of use of systematic forecasting methods.

**Fig 2:**
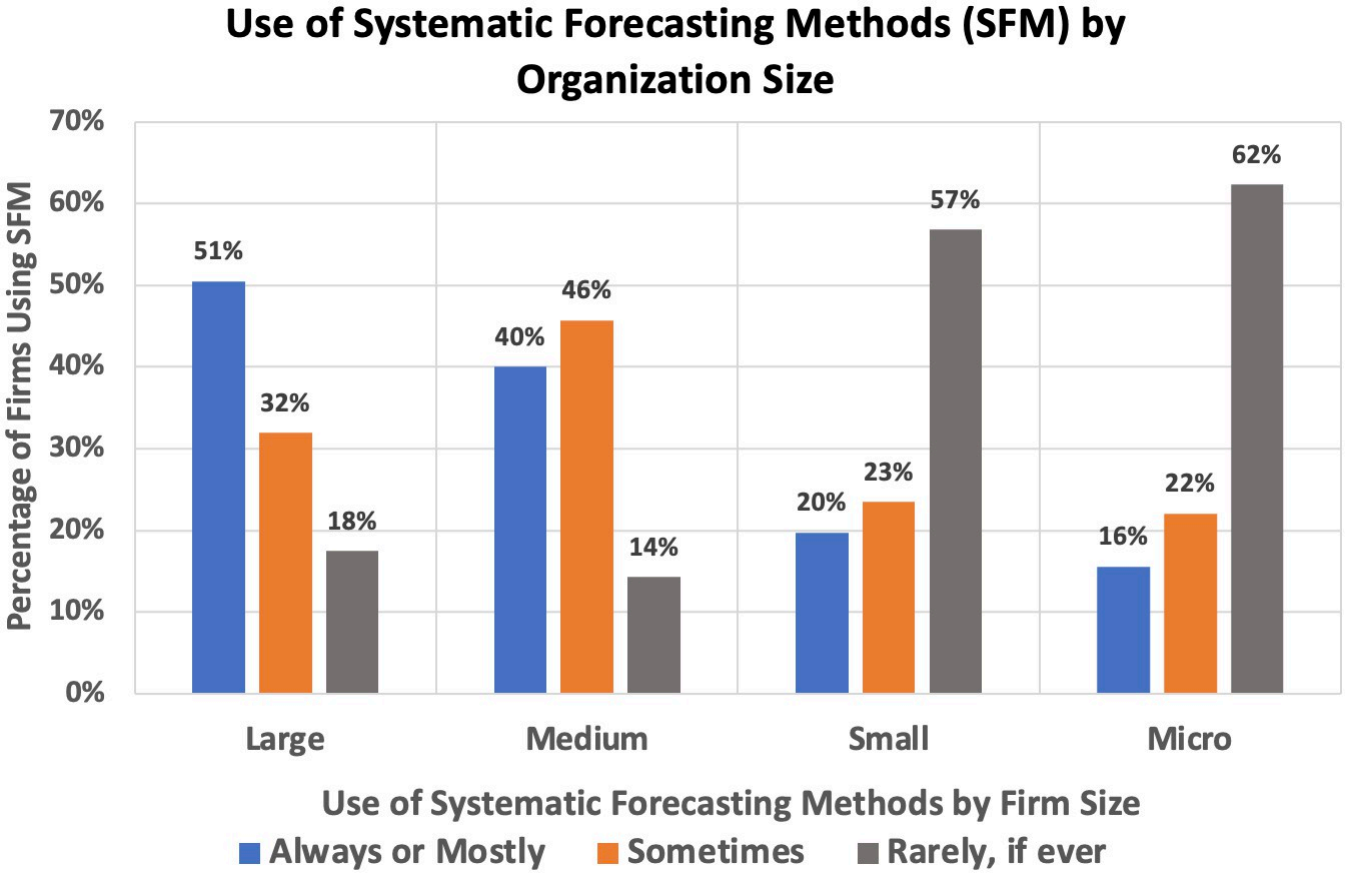
Use of systematic forecasting methods (SFM) by organization size.

Fig 1 shows only 28% indicated they “always” use systematic methods, while 44% answered “rarely, if ever”. Fig 2 shows the application of SFMs is more frequent in larger firms: 51% of managers in these organizations responded “always use” compared to only 16% in small or micro firms. Under 20% of large and medium firms reported “rarely, if ever”, while more than 50% of small and micro firms reported this.

However, while company size was a major determinant of SFM adoption, it was by no means the sole factor. Almost 20% of the large firms in the survey responded “rarely, if ever”, while about 40% of the small and micro firms report at least partial use.

We asked firms reporting “rarely, if ever” to identify the main reasons for none use. Responses were received from 92 of the 164 firms in this category.

1. Our firm is too small to engage a forecasting specialist or hire outside expertise. (52 responses)
2. We are unfamiliar with statistical/machine learning (ML) methods and software. (40 responses)
3. We are satisfied with the way we now make our projections. (28 responses)
4. We do not trust the results of commercial forecasting software packages. (11 responses)
5. We believe that statistical/ML methods are too difficult or costly to implement. (10 responses)
6. We do not need to forecast because we respond to customer orders as they arrive. (10 responses)
7. We think that statistical/ML forecasts will not be accurate enough to meet expectations. (5 responses)

The majority of responses reveal a lack of means, lack of familiarity with SFMs, and concerns about cost and accuracy. For these companies, proof that systematic methods can improve forecasting performance and be implemented at reasonable cost (without the requirement of specialist staff) might be persuasive. Nevertheless, many respondents expressed satisfaction with their current processes and there is evidence that suboptimal forecasting can persist simply because the existing process suits all parties involved [6]. Algorithm-based forecasts can deprive managers of a sense of ownership of forecasts and deny them the opportunity to demonstrate their market knowledge and expertise. Accordingly, software suppliers are happy to provide facilities for the easy, and often unjustified, overriding of algorithmic forecasts, while conveniently, the use of their software is suggestive of a scientific forecasting process.

There was also evidence of a lack of trust in forecasting. Trust is likely to arise from understanding how forecasts are derived and what they can achieve, together with a perception they are reliable [9]. Our interviews revealed that many forecast users, including senior managers, had unrealistic expectations of forecasting. They did not recognize that forecasting accuracy is constrained when outcomes are subject to high levels of noise. One company expected less than a 20% error when its demand history exhibited a largely random pattern.

Several interviewees suggested that managers’ expectations were often influenced by “glitzy” pitches from software vendors who made exaggerated claims for the accuracy their product could achieve. When these claims failed to materialize, managers switched to skepticism and disbelief in forecasting’s potential. Most forecast users, and even some forecast providers, lack a technical background, so forecasts created by the latest ML methods in particular pose an explainability problem that can also erode trust.

The interviews revealed additional reasons for the failure to employ systematic methods. Forecasts are often confused with targets, budgets and plans which are likely to be at odds with the output of SFMs. While a systematic forecast can provide a reliable basis for such activities, their conflation with forecasting can lead to processes dominated by judgment and organizational objectives so that a systematic forecast is not seen to have a useful role.

Organizational politics were also a major barrier and often produced incentives to distort forecasts. For example, demand forecasts were manipulated upwards to keep senior staff happy (“enforcing”) or kept artificially low for the sales unit to gain kudos when their forecasts were exceeded (“sandbagging”). In addition, forecasters were sometimes motivated to discourage the adoption of algorithms, fearing that automation could lead to the loss of their job. In some organizations, there was an adversarial relationship between departments that restricted the sharing of crucial information. Several interviewees blamed IT departments for refusing or being unable to supply data at the frequency forecasters required.

The personnel producing forecasts pose another barrier to SFM implementation: forecasters are not always trained in forecasting methods, and some viewed their job merely as a phase in their career and therefore not worthy of a significant investment in learning and self-development. Forecasters, according to one consultant, rarely felt pressured to perform the forecasting task to the best of their ability.

## 6 Departures from systematic forecasting methods

### 6.1 Judgmental interventions

An unfortunate implication of lack of trust in the output of forecasting software is the tendency for unwarranted judgmental interventions.

**Fig 3:**
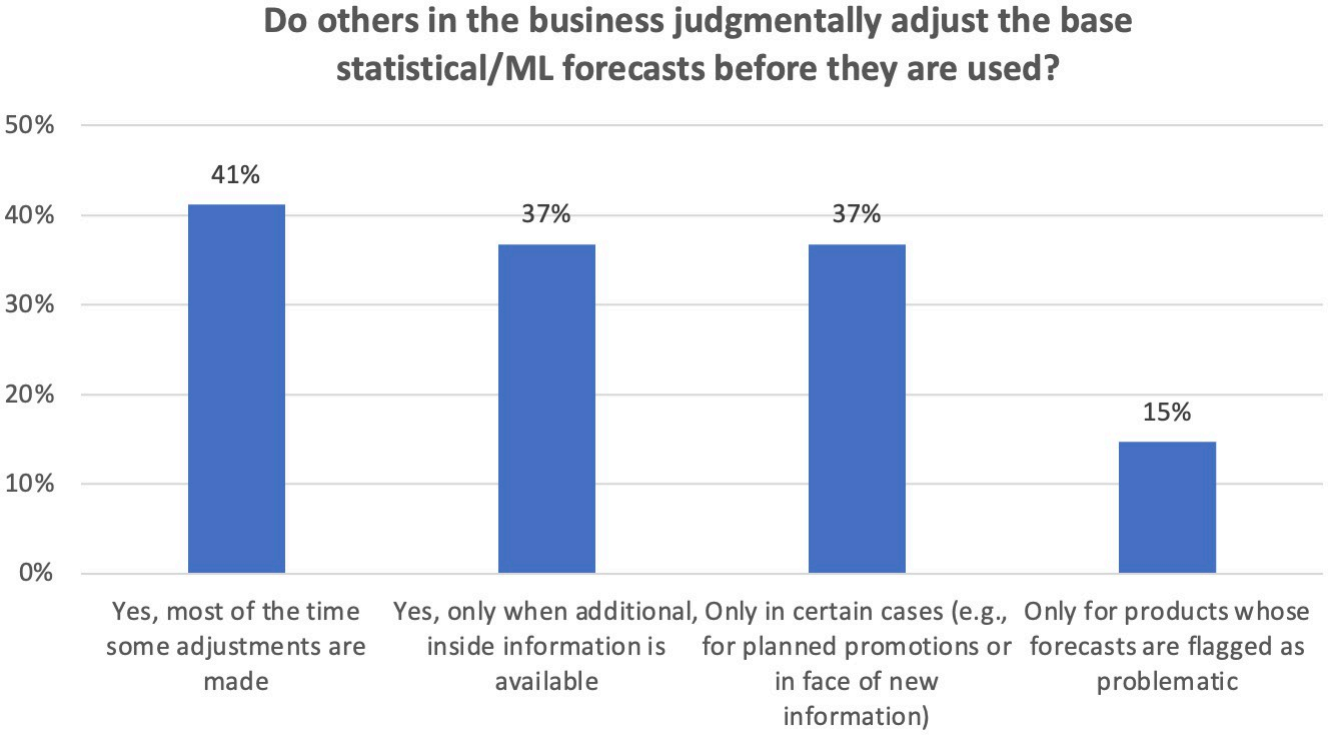
Extent of judgmental adjustments (n = 173 responses).

Fig 3 reveals that judgmental interventions were common in the firms we surveyed, a finding corroborated by our interviewees and suggesting a conflict with advice based on extensive research: namely, that such interventions should be made sparingly, where important and reliable new information becomes available that is not incorporated into the algorithm-based forecast. Research suggests that in practice many interventions are made because people mistakenly see systematic patterns in the random noise found in time series [10], or because they regard each forthcoming period as a unique case so that algorithmic forecasts based on past data are seen as irrelevant [11]. The need for a sense ownership of forecasts (see above) can also lead to excessive judgmental intervention. Our interviews revealed that interventions were often made *covertly*, by manipulating parameters in the algorithm or the length of the dataset until a desired forecast was reached.

Barely half the organizations surveyed analyze the forecast value added (FVA) [12] of judgmental adjustments, so that little is known of when or even whether adjustments tend to improve forecast accuracy. The potential gains of using FVA were revealed by a demand forecaster we interviewed at an American medical equipment company. He found that 2/3 of the adjustments were upwards and these generally increased errors, “massively destroying value.” In contrast, the 1/3 that were downwards tended to reduce errors significantly, a finding supported by research in other organizations [13].

### 6.2 Software

**Fig 4:**
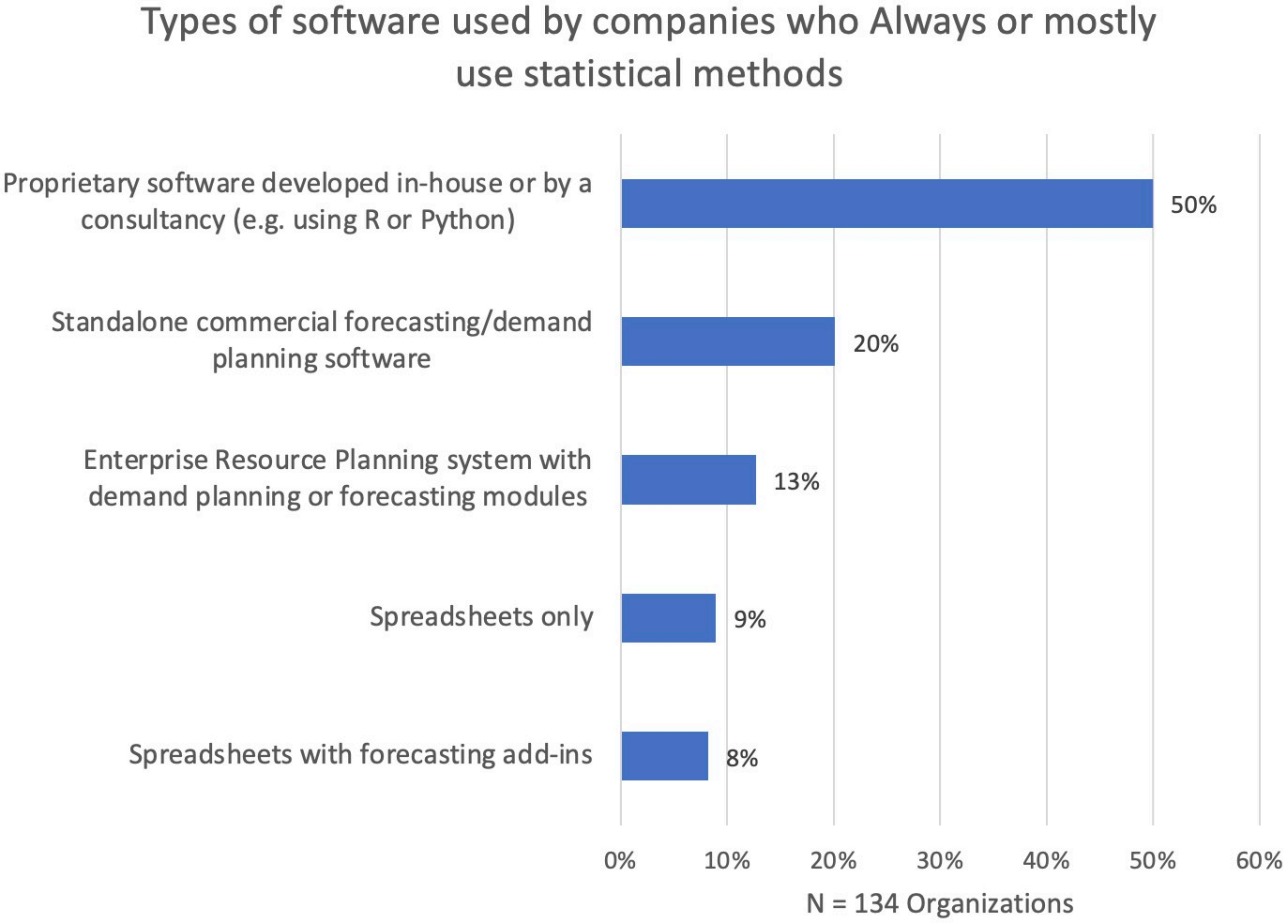
Types of software used by companies who always or sometimes use SFMs.

The interviews suggested persistent reliance on Microsoft Excel to generate forecasts, despite the limitations of spreadsheet software both in managing complex and interlinked worksheets and in the provision of adequate forecast modeling and evaluation capabilities. One consultant expressed concern that reliance on spreadsheets could propagate errors and contaminate forecasts. He attributed a firm’s preference for spreadsheets to the forecaster’s desire to maintain ownership and control over the forecasts. However, our survey (Fig 4) indicated that the sole use of spreadsheets was far less common in organizations that were employing SFMs.

Fig 4 also shows that dedicated commercial forecasting software is used by only 20% of the organizations. However, the interviews revealed that such software is not always up to date and may provide limited facilities, such as inability to model promotion spikes and outliers. In some software, there are severe restrictions on how forecasts in a product hierarchy are reconciled, such as permitting only bottom-up aggregation of forecasts made at the most granular level, a practice that works poorly in the very frequent situations when the granular data are short and noisy.

Half of the organizations that claimed to use SFMs employed proprietary software. However, the extent to which this embodies modern forecasting principles is unclear. In the interviews, we also learned of situations where company acquisitions led to the use of multiple, incompatible systems. There were reports of newly appointed senior managers purchasing “trendy” AI or ML software without basic knowledge of its potential and drawbacks, and without consultation of its forecasters.

### 6.3 Use of data

Only half of our surveyed firms maintained a database of historical sales, a critical precondition for application of statistical methods. The reasons reported included lack of resources but also the questionable belief that historical data were not useful for the business. Overcoming the data deficit should be a major component of initiatives to incentivize use of SFMs.

Our interviews elicited criticism of the handling of data, neglecting tasks such as data cleaning to remove errors/outliers, appropriate variable transformations, and reliance on recorded orders to produce demand forecasts instead of point-of-sale (POS) data from store scanners, which give a truer and faster signal of changing demand patterns.

### 6.4 Uncertainty measurement

**Fig 5:**
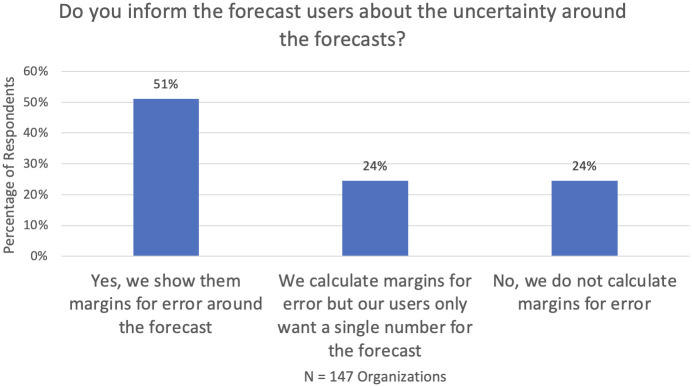
Reporting of uncertainty.

Our survey indicated that more than 90% of those organizations that *always or sometimes* use SFMs say they do evaluate and monitor forecast accuracy. But the value of doing so is often suspect. As shown in Fig 5, barely half convey the margins for error to their users, while about 25% do not bother to do the simple conversion of accuracy metrics into margins for error in the forecast.

Although uncertainty assessment is of the utmost importance in inventory management and service-level provision, our interviews suggested that formal consideration of uncertainty is scarce, even when their software produced prediction intervals. Explanations for this neglect included presumed lack of management interest or manager intolerance for wide prediction intervals, even though these represent a realistic assessment of the true level of uncertainty.

## 7 Paths to greater adoption of SFMs

Organizations should be more amenable to using SFMs if they can see clear benefits—particularly financial—from their adoption. Of course, some gains from systematic forecasting, such as reduced environmental impacts, are difficult to monetize, but many others such as inventory cost savings can be readily measured.

However, managers, particularly senior managers, need to be convinced that these benefits will justify the costs of investment in a systematic forecasting process. Several interviewees argued that the buy-in of senior management is critical: if managers are not championing a systematic forecasting process, it’s unlikely to be successfully implemented. Some methods, such as those rooted in machine learning, do require a considerable financial outlay for software and staff training. This commitment can seem particularly unattractive to managers who are skeptical about the value of forecasting or committed to methods that are explainable. But this is not to gainsay the prospect that many SFMs are based on simpler methods, the best of which is capable of being automatically selected, applied at great scale, and readily explained to nontechnical users.

As a basis for program development, our survey posed two questions. First: *If your firm rarely, if ever, applies systematic methods to generate forecasts, what potential benefits might prompt you to learn more about these methods and evaluate their value for your company?*

Responses were received from only 29 companies. The paucity of responses from the 164 firms in this category probably suggests a widespread lack of familiarity with systematic methods. Those who did respond pointed to improved accuracy and cost-effectiveness, better service to clients (internal and external), and greater influence on management. One respondent suggested that seeing an application of systematic methods in a similar context to their own would help. Another pointed out that, though systematic methods would help in planning some actions further ahead, it “would require organizational and process changes … a setup that may be difficult to implement.”

Many of these considerations can be demonstrated to companies through side-by-side comparisons of the forecasts from simpler systematic methods against those from the current processes in place. Currently, such comparisons are offered to prospective clients only by software vendors, whose methodology cannot be verified and whose sales pressure can be a hindrance to experimentation. A free Web-based tool from an independent source (such as the International Institute of Forecasters, to whom a proposal has been tendered by the authors) and utilizing a proper design of a forecast accuracy comparison [14] could catalyze interest in such comparisons. A serious caveat, however, is that the organization must be willing to undertake construction of a historical database, now absent in half of our nonusing firms.

Secondly, we also asked the 103 firms who said they always used systematic methods: *If you were asked to describe for another organization (that does not now use systematic forecasting) the major benefits your organization has reaped from its systematic forecasting process, what benefits would you point out?*

The dominant themes noted by respondents were improved accuracy, objectivity, honesty, consistency, and avoidance of judgmental biases, all leading to improved forecasting performance, more reliable measurement of uncertainty, and the downstream benefits to planning, operations, and decision making. Several referred to the benefits of identifying a baseline forecast that allowed discussion of how the future will be different or why judgment calls differ from the baseline. One respondent said it allowed quantification of risk exposure emanating from historical volatility or changes in demand drivers. Another argued that it exposed assumptions that could be improved. Importantly, automated forecasting freed up time for planners to work on other things.

These gains can apply to firms of all sizes, so where organizations are too small to employ specialist personnel or purchase expensive software, outsourcing the production of forecasts to specialist companies should be evaluated for cost-effectiveness.

### 7.1 The GAC case

In addition to side-by-side demonstrations, a firm can be referred to case studies that describe successful implementations of SFMs and the ensuing benefits received. One such example is Golden Artist Colors (GAC), an employee-owned speciality chemical (paint) manufacturer based in New Berlin, N.Y (see also NorthFind Management Case Studies: Golden Artist Colors; information provided by J. Karelse, CEO NorthFind Management, who led the forecasting training and implementation). The company of about 100 employees utilized a forecasting process that relied on periodic estimates from its sales team. However, once GAC became aware of systematic forecasting methods, it was concerned that the scale and effort required to build data stores and the perceived complexity of statistically driven methodologies would disqualify them from ever recognizing the benefits.

In fact, the cost and effort turned out to be far less burdensome in practice. With a single designated demand planner given training in demand-planning best practices, GAC was able to build an initial table of historical demand from sales records, thereafter adding to them monthly. Seven years later, systematic forecasting is a core component of GAC’s strategic and tactical planning processes. The firm reported it has realized decreased inventory levels by more than 15%, while maintaining service levels above 98%. The reduction in waste improved due to heightened forecast accuracy, and their cost of goods sold (COGS) decreased by 26.5%.

Interviewees reported similar potential benefits. A retailer stated that the resulting increases in accuracy would lead to a reduction of (*i*) unsold goods offered at a cheaper price, (*ii*) the write-off of unsold perishables, (*iii*) costs of transporting unsold items for redistribution to other stores, and (*iv*) stockouts and lost profits. An airport operator said that improved accuracy was associated with better capacity planning and a more reliable indication of where future capital investments should be made. A mass-transport operator said it would reduce the risk that stations would be closed because of unforeseen staff shortages. A bank consultant said that there would be less chance of serious errors in estimates of the reserves needed to cover losses. One interviewee argued that the adoption of more systematic methods would also improve the morale of the forecasting team.

### 7.2 Mentoring

We are encouraged by the potential for knowledge-transfer partnerships between “mentor firms” (users of SFMs) and “virgin firms.” Our survey responses reveal a willingness of users of SFMs to serve as mentors in providing motivation and general guidance rooted in their own experience. To use an expression attributed to the French economist Jean-Baptiste Say—that the supply of a good or service creates demand for that good or service—this offering by mentor firms could incentivize the participation of virgin firms.

Of course, an administrative process must be created. For this we would again look to the International Institute of Forecasters, whose mission statement makes such knowledge-transfer efforts a primary objective: To paraphrase closely: “The IIF is dedicated to developing and furthering the generation, distribution, and use of knowledge on forecasting by bridging the gap between theory and practice, and bringing together decision makers, forecasters, and researchers to improve the quality and usefulness of forecasting” (see also https://forecasters.org/about/us/).

## 8 Conclusions

Systematic forecasting can bring significant gains to organizations. Indeed, we are unaware of any case where an enterprise employing these methods suffered detrimental effects in relation to its key objectives such as profitability and high levels of customer service. Yet our study has provided evidence of widespread skepticism about the value of forecasting and lack of awareness of its scope and rationale among key decision makers. As a result, forecasting often has a Cinderella role: under-resourced, implemented by untrained and unmotivated staff, distorted by ad hoc judgments, and contaminated by political objectives. Expectations of poor forecasting performance therefore become self-fulfilling. Though our interviews revealed several examples of good practice and supportive senior managers, these cases were rare; and many interviewees—forecasters and consultants—revealed an underlying frustration with current forecasting practices.

In a volatile and uncertain world, systematic forecasting presents a vast untapped potential for organizations to enhance performance and achieve competitive advantage. Advanced technologies that improve accuracy and lower uncertainty are already available, as are the results of extensive research that has delivered insights into how management judgment and computer-based methods should be integrated for maximum effectiveness. The challenge is to make high-level decision makers aware of these developments and convince them of the many advantages that systematic forecasting can bring to a business.

## Supporting information

S1 File (BIB)
